# Association between health-related physical fitness and self-reported health status in older Taiwanese adults

**DOI:** 10.1186/s12877-022-02929-4

**Published:** 2022-03-21

**Authors:** Yan-Jhu Su, Chen-Te Hsu, Chyi Liang, Po-Fu Lee, Chi-Fang Lin, Hung-Ting Chen, Chien-Chang Ho

**Affiliations:** 1 Department of Gerontology, University of Massachusetts Boston, Boston, MA 02125 USA; 2 Department of Recreation and Sport Management, Shu Te University, Kaohsiung City, 82445 Taiwan; 3 Department of Economics, Shih Hsin University, Taipei City, 116 Taiwan; 4 Department of Leisure Industry and Health Promotion, National Ilan University, Yilan County, 260 Taiwan; 5 Department of Physical Education and Sport Science, National Taiwan Normal University, Taipei City, 106 Taiwan; 6 Physical Education Office, Ming Chuan University, Taipei City, 111 Taiwan; 7 Department of Physical Education, Fu Jen Catholic University, No. 510, Zhongzheng Rd., Xinzhuang Dist., New Taipei City, 24205 Taiwan; 8 Research and Development Center for Physical Education, Health and Information Technology, College of Education, Fu Jen Catholic University, New Taipei City, 24205 Taiwan; 9 Department of Kinesiology, National Tsing Hua University, Hsinchu City, 30014 Taiwan

**Keywords:** Physical fitness, Self-reported health status, Older adults, Taiwan, Cross-sectional study

## Abstract

**Background:**

Aging is an inevitable process of life development. These physical changes may cause a decline in the functional adaptability and health of older adults. This study aims to determine if an association exists between health-related physical fitness measurements and self-reported health status in older Taiwanese adults.

**Methods:**

A total of 22,389 Taiwanese adults aged 65 years or older were recruited as study participants. Demographic characteristics, life habits, anthropometric assessments, health-related physical fitness measurements, and self-reported health status from this dataset were analyzed using the chi-square test, one-way analysis of variance, and logistic regression analysis.

**Results:**

The results showed that there was significant association between back scratch and self-reported health status (excellent/good) (odds ratio [OR], 1.003; 95% CI 1.000–1.006) after adjusting potential confounders (gender, height, weight, body mass index, education, monthly income, marital status, smoking status, and chewing betel nuts). However, adjusted OR for unhealthy status (poor/very poor) significantly decreased for chair sit-and-reach test (OR 0.994, 95% CI 0.988–0.999).

**Conclusions:**

The present study reveals significant associations between health-related physical fitness measurements and self-reported health status in older Taiwanese adults. In particular, the waist-to-hip ratio could be involved in the cognitive process of one’s subjective health status, since individuals’ perception of their physical appearance affects their self-reported health. Future researches are suggested to investigate the causality between health-related physical fitness and subjective health status.

## Background

Aging is an inevitable process of life development. One characteristic of aging is the degradation of the human musculoskeletal system, which leads to a decrease in muscle mass and lower muscle function [1, 2]. These physical changes may cause a decline in the functional adaptability and health of older adults.

Recently, researchers have increased their focus on health and aging. According to the World Health Organization [3], “health is a state of complete physical, mental, and social well-being and not merely the absence of disease or infirmity.” Physical fitness is a multi-factor indicator. The basic measures of physical fitness include height, weight, body mass index (BMI), body function, medical history, body composition, and age. Numerous advanced measures are also used to assess physical fitness, including cardiorespiratory endurance, muscular endurance, muscular strength, and body flexibility [4–6].

Obviously, in the advanced measures used to assess physical fitness, musculoskeletal-related measurements account for the majority. Musculoskeletal health consists of three elements: muscular strength, muscular endurance, and flexibility [4]. If these elements cannot be maintained, the fitness of the musculoskeletal system may be damaged. Musculoskeletal interaction is the essential for body movement, so poor musculoskeletal health will directly affect the physical condition of the individual. Kell et al. [7] pointed out that the execution of daily tasks may become a challenge due to age. They also illustrated that the combination of resistance training and stretching to improve musculoskeletal health is related to enhanced health. In addition, numerous studies have established the relationships of general physical fitness with health status and quality of life [4, 8–10].

Sato et al. used multiple discriminant analysis to quantify the relationship between the health status and physical fitness of middle-aged and older adults (ages 30 to 60 years) [8]. Their results revealed a relationship between health status and physical fitness. Daily physical activity may positively affect the health of middle-aged and older people. However, few studies have mentioned the relationship between physical fitness measurement and self-reported health status. Instead, most researchers have used physician assessments to determine the health status of participants.

Sato et al. have explored the relationship between the health status and physical fitness of middle-aged and older men [9] and women [10] in Japan. The studies identified components of physical fitness that helped to improve and maintain health in different age groups and quantified the relationship of health status with physical fitness in middle-aged and older individuals. For example, they suggested that for men aged 50 to 59, maintaining a normal BMI and improving cardiorespiratory endurance and balance are effective. For men aged 60 to 69, maintaining and improving flexibility and paying attention to body fat percentage are most important [9]. In addition, for women between the ages of 50 and 69, maintaining the percentage of body fat within an appropriate range is most important [10].

When researchers want to compare the health status between groups, self-reported health indicators are commonly used (also known as self-rated health). Self-reported health is a comprehensive measure of individual’s overall health and can be regarded as the output of an individual’s physical function and subjective self-health awareness [11].

We found no relevant research on the relationship between health status and physical fitness in Taiwan. Determining factors related to self-reported health is very important for overall population health, especially due to the aging trend of the overall population [12]. Determining these factors can also help the government formulate relevant incentive policies promote the health of the people [11]. Therefore, this study aims to determine if an association exists between health-related physical fitness measurements and self-reported health status in older Taiwanese adults.

## Methods

### Study design and participants

We conducted a cross-sectional study to determine the associations between physical fitness components and self-reported health status in older Taiwanese adults. We reviewed data derived from the National Physical Fitness Survey Databases in Taiwan (NPFSD 2014–15) from Taiwan’s Sports Administration, a branch of the Ministry of Education. All participants were recruited using convenience sampling from 35 examination stations in 20 cities or counties in Taiwan; the detailed procedure has been described elsewhere [13–15]. Participants completed a questionnaire on their demographic characteristics and life habits, and we measured resting heart rate and blood pressure for safe preliminary screening before conducting physical fitness measurements. Finally, for this study, we assayed the questionnaire and physical fitness measurement data of 22,389 Taiwanese adults aged 65 years and older. The data was collected between October 2014 and March 2015. This study was approved by the Ethical Committee of Fu Jen Catholic University (FJU-IRB-C108088), and written informed consent was obtained from each participant.

### Data collection

Face-to-face interviews and physical examinations were completed by trained research assistants and nurses. The questionnaire data included demographic characteristics (i.e., age, gender, education, monthly income, and marital status), life habits (i.e., smoking, betel-nut chewing, and dieting), and self-reported health status. Anthropometric measurements including body weight, height, waist circumference (WC), and hip circumference (HC) were taken after the participants had removed their shoes and heavy clothes. Body weight was measured to the nearest 0.1 kg using a weighing scale. Body height was measured to the nearest 0.1 cm using a metal measuring tape attached to a wall with an acute-angled head piece; the participants stood against this vertical wall and wore no shoes. BMI was calculated as weight in kilograms divided by the square of height in meters. WC was measured (to the nearest 0.1 cm) by using a soft measuring tape at the level of the natural waist, which was identified as the level at the hollow molding of the trunk when the trunk was bent laterally. HC was measured (to the nearest 0.1 cm) at the level of the greater trochanter. The waist-to-hip ratio (WHR) was also calculated. The cut-off BMI values were those suggested by Taiwan’s Ministry of Health and Welfare: underweight (BMI < 18.5 kg/m^2^), normal (18.5 ≤ BMI < 24 kg/m^2^), overweight (24 ≤ BMI < 27 kg/m^2^), and obese (BMI ≥ 27 kg/m^2^) [16].

### Self-reported health status

Self-reported health status and obtained from the following questionnaire item: “In general, would you say your health is excellent, good, fair, poor, or very poor?” In this analysis, we have combined the responses into a ternary outcome, where self-reported health status could be health status (a response of “excellent” or “good”), fair (a response of “fair”), or unhealthy status (a response of “poor” or “very poor”), in order to concentrate the categories for further comparisons. This approach has been presented in the previous studies [13–15].

### Physical fitness measurements

To determine the functional capacity of older adults, we assessed five main components of physical fitness based on nine indicators: aerobic endurance (2-min step), muscle strength and endurance (30-s arm curl and 30-s chair stand), flexibility (back scratch and chair sit-and-reach), balance (one-leg stance with eyes open and 8-ft up-and-go), and body composition (BMI and WHR). Each physical fitness indicator had accompanying performance standards for male and female participants aged 65 years and older based on an annual national survey of more than 20,000 older Taiwanese adults. Measurements and above-mentioned data were recorded by examiners who had attended an official training seminar and passed a certification test on standardized procedures. The details of the 2-min step, 30-s arm curl, 30-s chair stand, back scratch, chair sit-and-reach, and 8-ft up-and-go were strictly performed according to the Senior Fitness Test manual [17], and the one-leg stance with eyes open was as described in other articles [18].

The content and the process of the physical fitness measurements were explained to the participants, and they had 10 min to warm up so that their optimum performance could be achieved. Measurements were scheduled before the exercises. All participates were assessed in the following order with sufficient rest (3 to 5 min) between measures: body weight, height, WC, HC, one-leg stance with eye open, 30-s chair stand, 30-s arm curl, 2-min step, chair sit-and-reach, back scratch, and 8-ft up-and-go. For each indicator, the participants were classified into four quartiles according to their physical fitness performance levels.

### Statistical analyses

The data were analyzed using the Statistical Analysis System (SAS) software package (Version 9.4, SAS Institute Inc., Cary, NC). Differences in demographic data and physical fitness measurements between categories of self-reported health status were analyzed using one-way analysis of variance (ANOVA) or chi-square tests. When a significant *F* value was found (*p* <  0.05), Tukey’s post hoc test was performed to determine the differences between the pairs of means. Logistic regression models were used to estimate the odds ratios (ORs) and 95% confidence intervals (CIs) for the association between quartiles of each physical fitness measurements and self-reported health status (excellent/good vs. fair and poor/very poor vs. fair) while adjusting for potential confounders. All data are expressed as means ± standard deviation or frequency (percentage). The significance level adopted to reject the null hypothesis was *p* <  0.05.

## Results

This study obtained data from 22,389 participants aged 65 years and older. The relationship between demographic characteristics and anthropometric indices of self-reported health status are shown in Table 1. Significant associations were identified between self-reported health status and all the demographic characteristics except age. Participants whose self-reported health status was excellent or good were the tallest (156.77 ± 7.92 cm) and heaviest (60.88 ± 9.75 kg).

**Table 1:** Demographic characteristics and anthropometric indices of study subjects according to self-reported health status

Variables	Self-perceived health status			*p*	Tukey’s post hoc test
	Excellent/Good	Fair	Poor/Very poor		
No. of subjects (%)	14,959 (66.81%)	5729 (25.59%)	1701 (7.60%)		
Age (y)	73.27 ± 6.48	73.32 ± 6.40	73.38 ± 6.06	0.713	
Gender (% men)	37.3%	33.4%	31.0%	< 0.001	
Height (cm)	156.77 ± 7.92	156.14 ± 7.67	155.82 ± 7.68	< 0.001	EG > F, PV
Body weight (kg)	60.88 ± 9.75	60.41 ± 9.95	60.84 ± 10.21	0.009	EG > F
Obese Status (%)				0.011	
Underweight	2.0%	2.8%	3.2%		
Normal weight	40.6%	40.5%	36.7%		
Overweight	33.7%	31.3%	31.4%		
Obese	23.7%	25.4%	28.6%		
Education (%)				< 0.001	
Elementary school or lower	57.7%	61.1%	68.9%		
Junior or senior school	28.2%	26.2%	21.8%		
College or higher	14.1%	12.8%	9.3%		
Monthly Income (%)				< 0.001	
≦ 20,000 NTD	87.6%	89.5%	93.0%		
20,001–40,000 NTD	7.3%	6.2%	3.5%		
≧ 40,001 NTD	5.1%	4.3%	3.5%		
Marital Status (%)				< 0.001	
Never married	56.0%	51.4%	53.4%		
Married	25.8%	26.8%	22.5%		
Divorced/widowed/other	18.1%	21.7%	24.1%	< 0.001	
Smoking Status (%)					
Never	92.5%	90.2%	89.5%		
Current	4.5%	5.6%	6.1%		
Former	3.0%	4.1%	4.4%		
Chewing betel nuts (%)				< 0.001	
Never	98.0%	97.2%	95.6%		
Current	0.9%	1.2%	2.0%		
Former	1.1%	1.7%	2.4%		

Values are expressed as means ± standard deviation

*Abbreviations*: *EG* Excellent/Good, *F* Fair, *NTD* new Taiwan dollar, *PV* Poor/Very poor

* *p* <  0.05

Table 2 compares the physical fitness measurements between self-reported health status categories. Significant associations were observed between self-reported health status and physical fitness. Both men and women whose self-reported health status was excellent or good had the highest levels of physical fitness (2-min step test, 30-s arm curl, 30-s chair stand, back scratch, chair sit-and-reach test, 8-ft up-and-go, and one-leg stance with eyes closed), but no significant association was identified between self-reported health status and BMI in men (*p* = 0.112).

**Table 2:** The comparison of functional fitness measurements according to self-reported health status

Variables	Self-perceived health status			*p*	Tukey’s post hoc test
	Excellent/Good	Fair	Poor/Very poor		
**Men** (*n*)	5580	1913	527		
2-min step test	87.40 ± 27.69	82.36 ± 28.11	76.24 ± 30.87	< 0.001	EG > F > PV
30-s arm curl	18.13 ± 6.27	17.41 ± 6.13	15.91 ± 5.96	< 0.001	EG > F > PV
30-s chair stand	15.62 ± 5.25	14.88 ± 5.27	13.26 ± 4.93	< 0.001	EG > F > PV
Back scratch	−10.86 ± 13.55	−12.04 ± 14.19	−15.00 ± 13.91	< 0.001	EG > F > PV
Chair sit-and-reach test	1.28 ± 10.82	0.57 ± 10.73	−2.08 ± 11.62	< 0.001	EG > F > PV
8-ft up-and-go	7.43 ± 2.52	7.84 ± 2.66	8.81 ± 3.23	< 0.001	EG < F < PV
One-leg stance with eye open	16.24 ± 11.44	15.37 ± 11.63	12.57 ± 11.29	< 0.001	EG > F > PV
BMI (kg/m^2^)	24.66 ± 3.03	24.77 ± 3.32	24.92 ± 3.45	0.112	EG < F < PV
WHR	0.92 ± 0.06	0.93 ± 0.06	0.93 ± 0.06	< 0.001	EG < F, PV
**Women** (*n*)	9379	3816	1174		
2-min step test	84.68 ± 27.25	81.09 ± 29.00	74.71 ± 32.10	< 0.001	EG > F > PV
30-s arm curl	17.62 ± 6.04	16.85 ± 5.93	16.01 ± 5.78	< 0.001	EG > F > PV
30-s chair stand	15.05 ± 4.89	14.25 ± 4.80	13.08 ± 4.82	< 0.001	EG > F > PV
Back scratch	−4.40 ± 11.08	−5.65 ± 11.60	−7.55 ± 12.65	< 0.001	EG > F > PV
Chair sit-and-reach test	5.41 ± 9.75	4.83 ± 9.80	3.67 ± 10.29	< 0.001	EG > F > PV
8-ft up-and-go	7.65 ± 2.42	8.03 ± 2.6	8.84 ± 2.99	< 0.001	EG < F < PV
One-leg stance with eye open	14.91 ± 11.16	13.85 ± 11.00	11.64 ± 10.45	< 0.001	EG > F > PV
BMI (kg/m^2^)	24.77 ± 3.50	24.74 ± 3.58	25.09 ± 3.78	0.001	EG, F < PV
WHR	0.88 ± 0.07	0.88 ± 0.07	0.89 ± 0.07	< 0.001	EG, F < PV

Values are expressed as means ± standard deviation

*Abbreviations*: *BMI* body mass index, *CEI* cardiorespiratory endurance index, *EG* Excellent/Good, *F* Fair, *PV* Poor/Very poor, *WHR* waist-to-hip ratio

*Significantly different males and females by one-way ANOVA at *p* <  0.05

Tables 3 and 4 represent the relationship between physical fitness and self-reported health status (excellent/good) after adjusting for potential confounders (gender, height, weight, BMI, education, monthly income, marital status, smoking status, and chewing betel nuts). Before adjusting, no significant associations were identified between five physical fitness indicators (back scratch: OR = 1.002, 95% CI 0.999–1.004; chair sit-and-reach test: OR = 1.002, 95% CI 0.999–1.005; one-leg stance with eye open: OR = 1.002, 95% CI 0.998–1.005; BMI: OR = 1.004, 95% CI 0.995–1.014; WHR: OR = 1.047, 95% CI 0.670–1.637) and self-reported health status (excellent/good). However, after adjustment, a significant association between the back scratch indicator and self-reported health status (excellent/good) was revealed (OR = 1.003; 95% CI 1.000–1.006). No significant relationships were observed between six physical fitness indicators (2-min step test: OR = 0.999, 95% CI 0.997–1.001; 30-s arm curl: OR = 0.992, 95% CI 0.982–1.003; back scratch: OR = 0.997, 95% CI 0.993–1.002; chair sit-and-reach test: OR = 0.995, 95% CI 0.989–1.001; BMI: OR = 1.012, 95% CI 0.995–1.028; WHR: OR = 1.010, 95% CI 0.449–2.273) and low self-reported health status (poor/very poor). However, once the results were adjusted for potential confounders, the ORs for self-reported health status (poor/very poor) decreased, and the correlation became significant for the chair sit-and-reach test (OR = 0.994, 95% CI 0.988–0.999).

**Table 3:** Multivariate adjusted ORs for health status (dependent variable) in relation to each functional fitness measurement after adjustment for potential confounders

	Factors not adjusted			Factors adjusted^a^		
	OR	95% CI	*p*	OR	95% CI	*p*
2-min step test	1.002	1.000–1.003	0.010	1.001	1.000–1.003	0.031*
30-s arm curl	1.007	1.001–1.013	0.014	1.007	1.001–1.013	0.016*
30-s chair stand	1.013	1.005–1.021	0.002	1.012	1.004–1.020	0.004*
Back scratch	1.002	0.999–1.004	0.253	1.003	1.000–1.006	0.031*
Chair sit-and-reach test	1.002	0.999–1.005	0.236	1.003	1.000–1.006	0.089
8-ft up-and-go	0.977	0.962–0.992	0.003	0.983	0.968–0.998	0.031*
One-leg stance with eye open	1.002	0.998–1.005	0.328	1.001	0.998–1.004	0.683
BMI (kg/m^2^)	1.004	0.995–1.014	0.391	0.967	0.885–1.056	0.454
WHR	1.047	0.670–1.637	0.841	0.744	0.469–1.183	0.212

*Abbreviations*: *BMI* body mass index, *CEI* cardiorespiratory endurance index, *CI* confidence interval, *OR* odds ratio, *WHR* waist-to-hip ratio

^a^Adjusted for gender, height, weight, BMI, education, monthly income, marital status, smoking status, and chewing betel nuts

* *p* <  0.05

**Table 4:** Multivariate adjusted ORs for unhealthy status (dependent variable) in relation to each functional fitness measurement after adjustment for potential confounders

	Factors not adjusted			Factors adjusted^a^		
	OR	95% CI	*p*	OR	95% CI	*p*
2-min step test	0.999	0.997–1.001	0.297	0.999	0.997–1.001	0.313
30-s arm curl	0.992	0.982–1.003	0.149	0.991	0.980–1.002	0.092
30-s chair stand	0.981	0.966–0.995	0.011	0.984	0.969–0.999	0.041*
Back scratch	0.997	0.993–1.002	0.202	0.996	0.991–1.001	0.090
Chair sit-and-reach test	0.995	0.989–1.001	0.076	0.994	0.988–0.999	0.030*
8-ft up-and-go	1.055	1.029–1.081	< 0.001	1.054	1.027–1.080	< 0.001*
One-leg stance with eye open	0.993	0.987–0.999	0.014	0.993	0.988–0.999	0.026*
BMI (kg/m^2^)	1.012	0.995–1.028	0.167	1.018	0.981–1.056	0.347
WHR	1.010	0.449–2.273	0.981	1.132	0.489–2.622	0.771

*Abbreviations*: *BMI* body mass index, *CEI* cardiorespiratory endurance index, *CI* confidence interval, *OR* odds ratio, *WHR* waist-to-hip ratio

^a^Adjusted for gender, height, BMI, education, monthly income, marital status, smoking status, and chewing betel nuts

* *p* < 0.05

## Discussion

In this study, we examine the relationship between self-reported health status and physical fitness measurement results. The results indicate that regardless of gender, participants who perceived their health status as good exhibited significantly higher physical fitness measurement results than those in the other two groups, who reported their health status as either fair or poor. Therefore, the present study indicates that people with good self-reported health status also perform higher in physical fitness.

In addition, body shape is a factor for woman that affects self-reported health status. Nonetheless, the relationship between BMI and self-reported health status was not significant in men (*p* = 0.112). However, for women in this sample, the relationship between BMI and self-reported health status was statistically significant (*p* <  0.001) which means the heavier weight (normal BMI) have the better self-reported health. According to the results, the BMI are significant different among different self-reported health group in women but not men. We conclude that this may be related to the fact that women are more concerned about body shape [19]. Generally speaking, women still feel obese when they are of normal weight. Therefore, this may cause women to reach statistical significance in the relationship between BMI and self-reported health status. The limitation of this study is that the unbalanced male to female ratio of participants of this data collection. This gender difference may be caused by the convenience sampling which the gender ratio subjects was 1:2 (male: female) of this convenience sample. Therefore, in future research design, we suggest increasing the sample size and controlling the gender ratio to examine gender differences in the association between BMI and self-reported health status. In addition, there were significant bivariate associations between WHR and general health among men and women (that did not hold in adjusted analyses). We suggest that WHR may be a superior predictor than BMI in the future research. Although BMI is a very intuitive indicator for the definition of obesity, not all individuals at risk for obesity-related diseases are identified [20]. Additionally, individuals with large muscle proportions will generally have a relatively high BMI. It is also unlikely to define these individuals as obese.

The advantage of this study is that it uses data collected by the NPFSD for Taiwan’s Sports Administration. This data comes from different counties and cities across Taiwan and is therefore representative. Thus, it eliminates the researcher’s sampling selection bias. Furthermore, according to the literature review, although there are numerous studies have established the relationships of general physical fitness with health status and quality of life [4], few studies have examined the correlation between physical fitness performance and health among older adults. Sato et al. examined the relationship between anatomical structure and the quality of life related to body function and health [21]. They found that by verifying physical fitness measurement, health status can be estimated with relative accuracy. Another study confirmed that different musculoskeletal components have a strong influence on health status and quality of life which suggested that providing interventions for the older adults with high body weight, low lower limb strength, poor lumbosacral flexion ability and poor balance ability can improve their health and quality of life [4].. In this analysis, we examine the association between physical fitness and self-reported health status. The cross-sectional study design was also a limitation of this study. We cannot confirm the causality between these two variables. Therefore, we can continue to examine whether living habits may be mediating or moderating variables. In addition, different physical fitness measures could also be compared in future studies.

Evidence clearly supports the notion that exercise contributes to healthy aging. Angevaren et al. [22] concluded that aerobic physical exercise that improves cardiorespiratory health is beneficial to adults’ cognitive function. However, in their research, most of the comparisons results were not statically significant. In addition, with regard to improving the health of older adults, healthcare providers must take into account appropriate physical exercises to maintain and improve the level of physical health [23]. Research has also shown that physical activity improves the health of older adults [24]. To gain a more comprehensive understanding of the factors affecting health, we suggest that future studies include surveys of older adults’ exercise routines. Finally, due to a government provided questionnaire was conducted, the measurement of subjective health status may be insufficient which is also a limitation of this study. The self-reported health scale, such as SF-12, SF-36, should be applied for the future studies [4]. Also, quality of life survey is recommended to take part in the analysis in order to distinguish the dimensions of health status. In future research, we suggest that a more comprehensive self-reported health status survey be provided to obtain more information.

## Conclusion

In conclusion, although the effect sizes are finite in relevant comparisons, the present study reveals significant associations between health-related physical fitness measurements and self-reported health status in older Taiwanese adults. In particular, the WHR could be involved in the cognitive process of one’s subjective health status. WHR can be considered an indicator of physical appearance [19]. Therefore, individuals’ perceptions of their physical appearance affect their scores on self-reported health. Future researches are suggested to investigate the causality between health-related physical fitness and subjective health status.

## Data Availability

The data that support the findings of this study are available from [the Sports Cloud: Information and Application Research Center of Sports for All, Sport Administration, Ministry of Education in Taiwan] but restrictions apply to the availability of these data, which were used under license for the current study, and so are not publicly available. Data are however available from the authors upon reasonable request and with permission of [the Sports Cloud: Information and Application Research Center of Sports for All, Sport Administration, Ministry of Education in Taiwan].

## Abbreviations

ANOVA: Analysis of variance
BMI: Body mass index
CI: Confidence interval
HC: Hip circumference
OR: Odds ratio
NTD: New Taiwan Dollar
SAS: Statistical Analysis System
WC: Waist circumference
WHO: World Health Organization
WHR: Waist-to-hip ratio
